# Prevalence, maternal characteristics, and birth outcomes of preeclampsia: A cross-sectional study in a single tertiary healthcare center in greater Kuala Lumpur Malaysia

**DOI:** 10.3389/fpubh.2022.973271

**Published:** 2022-10-17

**Authors:** Rosnah Sutan, Nurul Afzan Aminuddin, Zaleha Abdullah Mahdy

**Affiliations:** ^1^Community Health Department, Faculty of Medicine, Universiti Kebangsaan Malaysia, Bangi, Malaysia; ^2^Obstetrics and Gynecology Department, Faculty of Medicine, Universiti Kebangsaan Malaysia Medical Centre, Bangi, Malaysia

**Keywords:** preeclampsia, maternal characteristics, Malaysian, gestational hypertension, birth outcome

## Abstract

**Background:**

Preeclampsia is associated with an increased risk of adverse maternal and perinatal outcomes. This study aimed to assess preeclampsia prevalence in a Malaysian referral maternity hospital and the association between preeclampsia and maternal characteristics and outcomes.

**Methods:**

A cross-sectional study was conducted between January 2010 and December 2020 using secondary data from a single tertiary healthcare center in Greater Kuala Lumpur, Malaysia. A total of 40,212 deliveries were included for analysis to investigate the association between conditions (maternal characteristics and adverse birth outcomes) and preeclampsia. Multivariable logistic regression was conducted to assess the association between multiple independent variables and the outcome variable (preeclampsia).

**Results:**

The reported prevalence of preeclampsia was 1.6%. Pregnant women with preeclampsia had a higher risk of preterm delivery (67.7%), instrumental and cesarean delivery (74.7%), neonatal low birth weight (48.5%), neonatal 5-min Apgar score <7 (18.1%), and neonatal intensive care unit (NICU) admission (19.8%). There were significantly higher odds of developing preeclampsia among nullipara [adjusted odd ratio (adjOR) 1.792, 95% CI: 1.518–2.115], women with a previous history of preeclampsia (adjOR 5.345, 95% CI: 2.670–10.698) and women with multiple pregnancies (adjOR 1.658, 95% CI: 1.071–2.566). However, there is a significant association between maternal characteristic variables. There was a significant association when a combination of variables for risk assessment: the presence of anemia and gestational hypertension effect on preeclampsia (OR 26.344, 95% CI: 9.775–70.993, *p* < 0.002) and gestational hypertension without anemia on preeclampsia (OR 3.084, 95% CI: 2.240–4.245, *p* < 0.001). Similarly, an association was seen between chronic hypertension and younger age (<35 years old) on preeclampsia (OR 14.490, 95% CI: 9.988–21.021, *p* < 0.001), and having chronic hypertension with advanced maternal age (≥35 years old) on preeclampsia (OR 5.174, 95% CI: 3.267–8.195, *p* < 0.001). Both conditions had increased odds of preeclampsia, in varying magnitudes. Overall, the significant interaction effects suggest that a history of chronic or gestational hypertension has a different relationship to the incidence of preeclampsia depending on the maternal age and anemia status. Pregnant women with preeclampsia had significantly higher odds for preterm delivery (adjOR 6.214, 95% CI: 5.244–7.364), instrumental and cesarean delivery (adjOR 4.320, 95% CI: 3.587–5.202), neonatal low birth weight (adjOR 7.873, 95% CI: 6.687–9.271), 5-min Apgar score <7 (adjOR 3.158, 95% CI: 2.130–4.683), and NICU admission (adjOR 8.778, 95% CI: 7.115–10.830).

**Conclusions:**

Nulliparity, previous history of preeclampsia, and multiple pregnancies were associated with an increased risk of preeclampsia. The presence of different underlying conditions, such as chronic hypertension, anemia, and extremes of maternal age played an important role in increasing preeclampsia risk in the considered study. Larger samples are needed to validate such findings.

## Introduction

Preeclampsia occurs in 1–5% of all pregnancies and is responsible for significant maternal and perinatal morbidity and mortality (1–5). Approximately 8.5 million women are diagnosed with preeclampsia every year (6). Worldwide, preeclampsia is responsible for >500,000 fetal and neonatal deaths and >70,000 maternal deaths annually (5, 7). Thus, preeclampsia represents a significant threat to women's health.

Early recognition and early intervention in women with high-risk factors for preeclampsia are critical for preventing preeclampsia and its related complications (8, 9). Primary health clinics provide antenatal care, playing an important role in early detection and prevention strategies for managing preeclampsia and its complications (8). Furthermore, in Malaysia, we provide pre-pregnancy care, including preconception counseling, effective contraception, underlying comorbid disease control, and treatment adjustment prior to conception, which are important measures for women at risk of preeclampsia (10).

The Malaysian Clinical Practice Guidelines for managing hypertensive disorder in pregnancy recommend screening and identifying women at risk of preeclampsia for close surveillance and commencement of prophylaxis during antenatal visits (10, 11). In Malaysia, the current routine preeclampsia screening classifies pregnant women as a moderate risk if they are primigravid, aged >35 years, carrying multiple pregnancies, have a pregnancy interval of >10 years, have a body mass index (BMI) >35 kg/m^2^, or have a family history of preeclampsia (10, 11). Pregnant women are classified as high risk if they have at least one high-risk factor, such as hypertensive diseases during a previous pregnancy, chronic kidney disease, autoimmune disease [systemic lupus erythematosus or antiphospholipid syndrome, type 1 or type 2 diabetes mellitus (DM), or chronic hypertension] (10). This risk stratification is adapted from internationally recognized guidelines (10, 12, 13).

Despite abundant research on preeclampsia (14–16), the multiple ethnicities and diverse regional cultures, rapid socioeconomic and demographic transition, and a higher prevalence of obesity, unhealthy lifestyles, and non-communicable diseases (NCDs) in Malaysia may contribute to significant differences in the maternal risk factors for preeclampsia among Malaysian women (17–19). The incidence of preeclampsia varies by type of healthcare facility as almost all preeclampsia cases were managed at the tertiary care level and remain a major contributor to maternal deaths (10, 11, 20). Hence, the present study aimed to identify preeclampsia prevalence and the association between maternal characteristics and preeclampsia and assess preeclampsia birth outcomes in an urban tertiary healthcare center.

## Materials and methods

### Study design and setting

This study was conducted using secondary data from the Hospital Chancellor Tunku Muhriz (HCTM) in Greater Kuala Lumpur, Malaysia. The database was collected from year 2010 until 2020 involving pregnant women who delivered at HCTM. The hospital receives referral cases from district hospitals and both public and private clinics from nearby coverage areas. HCTM has 88 beds distributed in 4 obstetrics and gynecology wards and 9 obstetrics beds in a labor room with an average of 5,573 births per year that cater for Malaysian and non-Malaysian citizen. The maternal health records of all delivered mothers were entered in ObsCentral software maintained by the MedicLink Systems (M) Pvt Ltd (20).

### Samples

Birth data were recorded in both manual and electronic data records system (ObsCentral). The data captured by the electronic system (ObsCentral) was entered after patient has delivered. Our study used retrospective secondary data from the ObsCentral dataset which covered variables on maternal demographics, obstetric information, neonatal profiles, and birth outcomes. Birth outcomes that were reported as compulsory items were birth weight, Apgar score, neonatal intensive care unit (NICU) admission, and stillbirth. Neonatal profiling of genetic and chromosomal abnormalities, growth restriction and physical abnormalities were not routinely recorded in the electronic data system. Maternal medical history, comorbidity, and previous obstetric history were also not recorded in the electronic system but were documented in the manual case records (maternal handheld antenatal record books and hospital medical records). Data were entered into the electronic system by nurses, based on the admission sheet during patient admission to the HCTM labor room. All data were verified by senior medical officers.

The study received ethics approval from the Ministry of Health Malaysia (NMRR-19-1104-46049) (IIR) and the UKM Ethics Committee (FF-2019-371). The target population comprised pregnant women who delivered at HCTM in the period 2010–2020. We included pregnancies of 20 weeks' gestation, livebirths, and newborns with birth weights ≥500 g. We excluded non-citizens and women with incomplete data. A total of 40,212 deliveries were included for secondary data analysis.

### Outcome measure

The outcome measure was preeclampsia as defined by the International Society for the Study of Hypertension in Pregnancy (7): gestational hypertension accompanied by at least one of the following new-onset conditions at or after 20 weeks' gestation: proteinuria or maternal organ dysfunction (including acute kidney injury, altered liver function, neurological complications, hematological complications or uteroplacental dysfunction).

### Definition of variables

The dependent variables were classified as preeclampsia when cases retrieved from ObsCentral dataset were recorded as preeclampsia in the diagnosis section. We cross checked other information available for the case in the records to confirm the diagnosis: blood pressure of 140 mmHg systolic and/or 90 mmHg diastolic after 20 weeks of gestation, and presence of proteinuria or any other maternal organ dysfunction or fetal growth restriction. Cases without preeclampsia were cross checked for blood pressure of <140 mmHg systolic and/or <90 mmHg diastolic.

The independent variables included were maternal age group (teenager ≤19 years, advanced maternal age ≥35 years, 20–34 years), ethnicity (Malay, Chinese, Indian, other), antenatal risk level as classified according to the Malaysian color coding system (21) based on the Ministry of Health Malaysia risk assessment checklist (red color indicates very high risk; yellow for high risk; green for medium risk; white II for low risk; white I for very low risk), gravidity (gravida ≥6, gravida 1, gravida 2–5), parity (para ≥5, nulliparity, para 1–4), anemia (hemoglobin <11 g/dl) (Yes or No), multiple pregnancy (Yes or No), DM (Yes or No), gestational DM (GDM, Yes or No), heart disease (Yes or No), kidney disease (Yes or No), autoimmune disease (Yes or No), history of preeclampsia (Yes or No), history of gestational hypertension (Yes or No), gestational hypertension (Yes or No), chronic hypertension (Yes or No), and family history of hypertension (Yes or No). The fetal and maternal outcome variables were route of delivery [No: spontaneous vaginal delivery (including instrumental) or Yes: cesarean section], low birth weight (Yes: <2.5 kg or No: ≥2.5 kg), preterm delivery (<37weeks) or term), 5-min Apgar score (<7 or ≥7), and NICU admission (Yes or No).

### Data analysis

The data were analyzed using the IBM Statistical Package for the Social Sciences (SPSS) 22 as descriptive, and bivariate analysis was followed by multivariable logistic analysis. Data exploration during descriptive analysis was performed to assess data distribution, missing data distribution, and outliers. Missing value analysis was done using the SPSS Little MCAR (missing completely at random) test with the Expectation-Maximization function that revealed data missing completely at random. Missing data and outlier data were excluded from the data analysis. The assumptions to use binary logistic regression including independent observations, no perfect multicollinearity and linearity were assessed. The model produces ORs, which suggest increased, decreased or no change in odds of being preeclampsia as the outcome. Further analysis involved complete case analysis.

For categorical data, the sample characteristics are presented as the frequency and percentage. Simple logistic regression and the Pearson chi-square were used for bivariate analysis. Further analyses were conducted using multivariable logistic regression using steps as follows:

Step 1: Data exploration (Descriptive Statistics).

Step 2: Simple Logistic Regression.

Step 3: Variable selection & checking “linearity in the logit” (Preliminary main-effect model).

Step 4: Checking interaction & multicollinearity (Preliminary final model). Checking Interactions process was performed: checking all possible 2-ways interactions, create interaction terms, add into the preliminary main effect model as additional independent variable before running the model using “enter.” If an interaction term is significant (*P* < 0.05), the appropriate model is the main effect variables plus the significant interaction term. Bonferroni's correction is performed for the multiple testing issue adjustment. We checked one interaction term at a time for all significant variable in final model and found that maternal age and chronic hypertension, anemia and gestational hypertension have a significant interaction. Therefore, the final model includes maternal age (>35), parity, anemia, multiple pregnancy, history of preeclampsia, gestational hypertension and chronic hypertension with significant interaction variables. All these variables are known risk factors for preeclampsia (22) and were all adjusted for in the multivariable regression models.

Variables with a *P*-value of <0.2 were included for multivariable logistic regression. Presence of the interaction with large main effects (*p* < 0.01) were reported. Missing data and outlier data were excluded from data analysis. All *p*-values resulted from two-sided statistical tests. *P* < 0.05 was considered statistically significant. The Bonferroni correction was calculated for adjustment made to *P*-value by dividing critical *P*-value (*p* = 0.05) by four comparisons done, in order to reduce chances of false-positive results when multiple pair wise tests performed (23).

## Results

### Maternal characteristics

**Figure 1:**
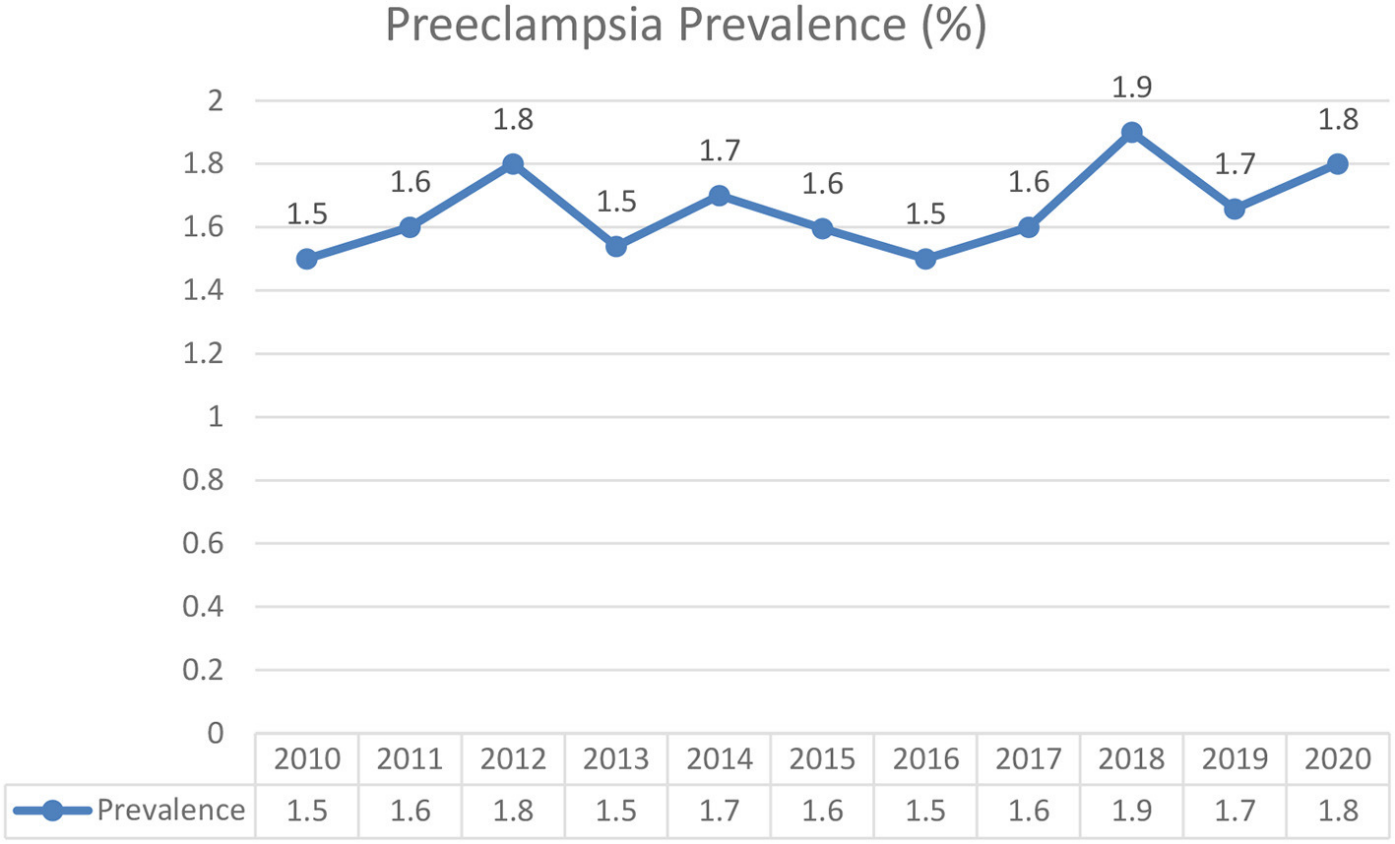
Prevalence of preeclampsia from 2010 to 2020 in Hospital Chancellor Tengku Mukhriz (HCTM).

Preeclampsia was present in 641 of 40,212 total deliveries (1.6%). The annual prevalence for year 2010 to 2020, were 1.55, 1.6, 1.8, 1.5, 1.7, 1.6, 1.9, 1.7, and 1.8% respectively (Figure 1). Table 1 shows the descriptive statistics of the maternal characteristics. Most patients were aged between 20 and 34 years, with 0.7% teenage pregnancy and 19.5% advanced maternal age. Around one-third who developed preeclampsia were of advanced maternal age (30.3%) compared to those who did not (19.4%). Most patients were Malay (68.6%). A total of 89.7% of the patients were tagged as white considering the Malaysian Color-Coding System for antenatal risk assessment (21), indicating a very low risk pregnancy. Preeclamptic women were noted among primigravidae (1.6%), women with multiple pregnancy (3.4%), anemia (3.7%), DM (1.9%), Gestational DM (12.8%), history of preeclampsia (1.7%), gestational hypertension (7.8%), and chronic hypertension (9.7%).

**Table 1:** Maternal sociodemographic, obstetrics and clinical characteristics.

**Variables**	**Preeclampsia**			***P*-value**
	**Yes *n* (%)**	**No *n* (%)**	**Total *n* (%)**	
	641 (1.6)	39.571 (98.4)	40.212 (100)	
**Age groups**				<0.001
<19 years old (Teenage pregnancy)	1 (0.1)	280 (0.7)	281(0.7)	
20–34 years 0ld	446 (69.6)	31,632 (79.9)	32,078 (79.8)	
More than 35 years old (advance maternal age)	194 (30.3)	7,659 (19.4)	7,853 (19.5)	
**Ethnicity**				0.921
Malay	435 (67.9)	27,142 (68.6)	27,577 (68.6)	
Chinese	91 (14.2)	5,438 (13.7)	5,529 (13.7)	
India	16 (2.5)	852 (2.2)	868 (2.2)	
Others	99 (15.4)	6,139 (15.5)	6,238 (15.5)	
**Antenatal risk#**				0.952
White 1	187 (29.2)	11.621 (29.4)	11.808 (29.4)	
White II	391 (61.0)	23.860 (60.3)	24,251 (60.3)	
Green	32 (5.0)	2.237 (5.7)	2,269.0 (5.6)	
Yellow	10 (1.6)	651 (1.6)	661 (1.6)	
Red	21 (3.3)	1,202 (3.0)	1,223 (3.0)	
**Gravida classification**				<0.001
Gravida 2–5	256 (39.9)	12.679 (32.0)	12,935 (32.2)	
Gravida 6 and more	349 (54.4)	25.236 (63.8)	25,585 (63.6)	
Gravida 1	36 (5.6)	1,656 (4.2)	1,692 (4.2)	
**Parity classification**				<0.001
1–4	301 (47.0)	14,701 (37.2)	15,002 (37.3)	
Para 5 and more	330 (51.5)	24,275 (61.3)	24,605 (61.2)	
Nulliparous	10 (1.6)	595 (1.5)	605 (1.5)	
**Anemia**				<0.001
Yes	24 (3.7)	5.137 (13.0)	5,161 (12.8)	
No	617 (96.3)	34.434 (87.0)	35,051 (87.2)	
**Multiple pregnancy**				0.011
Yes	22 (3.4)	795 (2.0)	817 (2.0)	
No	619 (96.6)	38.776 (98.0)	39,395 (98.0)	
**Diabetes mellitus**				<0.001
Yes	12 (1.9)	213 (0.5)	225 (0.6)	
No	629 (98.1)	39.358 (99.5)	39,987 (99.4)	
**Gestational DM**				0.262
Yes	82 (12.8)	5,681 (14.4)	5,763 (14.3)	
No	559 (87.2)	33.890 (85.6)	34,449 (85.7)	
**Heart disease**				0.930
Yes	1 (0.2)	100 (0.3)	101 (0.3)	
No	640 (99.8)	39.471 (99.7)	40,111 (99.7)	
**Kidney disease**				0.910
Yes	1 (0.2)	55 (0.1)	56 (0.1)	
No	640 (99.8)	39.516 (99.9)	40,156 (99.9)	
**Autoimmune disease**				0.302
Yes	3 (0.5)	80 (0.2)	83 (0.2)	
No	638 (99.5)	39.491 (99.8)	40,129 (99.8)	
**History of preeclampsia**				<0.001
Yes	11 (1.7)	81 (0.2)	92 (0.2)	
No	630 (98.3)	39.490 (99.8)	40,120 (99.8)	
**History of gestational hypertension**				
Yes	2 (0.3)	51 (0.1)	53 (0.1)	0.472
No	639 (99.7)	39.520 (99.9)	40,159 (99.9)	
**Gestational hypertension**				<0.001
Yes	50 (7.8)	850 (2.1)	900 (2.2)	
No	591 (92.2)	38.721 (97.9)	39,312 (97.8)	
**Chronic hypertension**				<0.001
Yes	62 (9.7)	384 (1.0)	446 (1.1)	
No	579 (90.3)	39,187 (99.0)	39.766 (98.9)	

#antenatal risk level is classified according to the Malaysian Color-Coding System (Ministry of Health antenatal risk assessment system: red, very high risk; yellow, high risk; green, medium risk; white II, low risk; white I: very low risk) (21).

### Factors associated with preeclampsia

Table 2 presents a simple logistic regression analysis, which indicated that pregnant mothers who were aged ≥35 years, nulliparous, had multiple pregnancy, DM, history of preeclampsia, gestational hypertension, or chronic hypertension, had significantly higher crude odd ratios (OR >1.00) for developing preeclampsia. However, anemic pregnant women had significantly lower crude ORs for developing preeclampsia (crude OR = 0.26; 95% CI: 0.173; 0.392).

**Table 2:** Factors associated with preeclampsia.

**Variable**	**Crude OR**	**95% CI**	***P***	**Adj. OR**	**95% CI**	***P***
Age groups			<0.001			<0.001
≥35 years old (Advance maternal age)	1.796	(1.515; 2.130)	<0.001^b^	2.075	(1.715; 2.511)	<0.001
≤19 years old (Teenage pregnancy)	0.253	(0.035; 1.809)	0.171^b^	0.214	(0.030; 1.527)	0.114
20–34 years Old	1.000			1.000		
Gravida classification			<0.001			
Gravida 6 and more	1.572	(1.111; 2.223)	0.011			
Gravida 1	1.460	(1.241; 1.718)	<0.001			
Gravida 2–5	1.000					
Parity classification			**<0.001**			0.001
Para 5 and more	1.236	(0.656; 2.331)	0.512	0.876	(0.458; 1.674)	0.479
Nulliparous	1.506	(1.287; 1.763)	**<0.001**	1.792	(1.518; 2.115)	<0.001
1–4	1.000			1.000		
**Anemia**
Yes	0.261	(0.173; 0.392)	<0.001	0.299	(0.198; 0.450)	<0.001
No	1.000			1.000		
**Multiple pregnancy**
Yes	1.734	(1.127; 2.667)	0.021	1.658	(1.071; 2.566)	0.034
No	1.000			1.000		
**Diabetes mellitus**
Yes	3.525	(1.960; 6.339)	<0.001			
No	1.000					
**History of preeclampsia**
Yes	8.512	(4.512; 16.058)	<0.001	5.345	(2.670; 10.698)	<0.001
No	1.000			1.000		
**Gestational hypertension**
Yes	3.854	(2.865; 5.184)	<0.001	3.482	(2.575; 4.709)	<0.001
No	1.000			1.000		
**Chronic hypertension**
Yes	10.928	(8.255; 14.46)	<0.001	8.999	(6.719; 12.053)	<0.001
No	1.000			1.000		
Age*chronic hypertension						0.002
≥35 years old* chronic hypertension				0.390	(0.217; 0.702)	0.002
Anemia*gestational hypertension				7.716	(2.764; 21.544)	0.001

Final model using Forward Stepwise (Likelihood Ratio); Hosmer and Lemeshow Test 0.292; Nagelkerke R Square 6.4%; Classification table 98.4%. *Interaction between variables. ^*b*^Wald test. The bold value means significant (*p* < 0.05).

Binary logistic regression examined whether the independent variables helped to explain occurrence of preeclampsia in a sample of 40,212 women. The sample included 641 women with preeclampsia and 39,371 women without preeclampsia. The data met the binary logistic regression assumptions of independent observations and no perfect multicollinearity. The multivariable logistic regression model analysis determined that maternal age ≥35 years, nulliparity, multiple pregnancy, history of preeclampsia, gestational hypertension and chronic hypertension were significantly associated with preeclampsia.

### Combination of variables for risk assessment

Table 3 depicts the results of selected analysis models for variables with significant interaction. The interactions between gestational age with chronic hypertension, and anemia with gestational hypertension, were significant factors associated with preeclampsia based on two dummy variables. Pregnant women with advanced maternal age (≥35 years old) without chronic hypertension, showed double the odds (odds 2.023; 95% CI: 1.673–2.477, *p* < 0.001) of developing preeclampsia compared to maternal age <35 years. Among the pregnant women with chronic hypertension, maternal age did not significantly increase the odds of developing preeclampsia. However, among maternal age <35 years with chronic hypertension showed an increased their odds of developing preeclampsia 14-fold (odds 14.490; 95% CI: 9.988–21.021, *p* < 0.001) compared to maternal age <35 years without chronic hypertension. In addition, among advanced maternal age (≥35 years), chronic hypertension increased their odds of developing preeclampsia 5-fold (odds 5.174; 95% CI: 3.267–8.195, *p* < 0.001) compared to maternal age ≥35 years without chronic hypertension.

**Table 3:** Selected model analysis for preeclampsia factors with interaction.

**Variable**	***N***	**Odds ratio**	**95% CI**	**Wald (*df*)**	***P*-value**
**Without chronic hypertension**
≥35 years	7,644	2.023	(1.673; 2.447)	52.702 (1)	<0.001
<35years	3,2122	1.000			
**Chronic hypertension**
≥35 years	209	0.663	(0.376; 1.168)	2.024 (1)	0.155
<35 years	237	1.000			
**Maternal aged** ** <35 years**
Chronic hypertension	237	14.490	(9.988; 21.021)	198.369 (1)	<0.001
Without chronic hypertension	31.841	1.000			
**Maternal aged** **≥35 years**
Chronic hypertension	209	5.174	(3.267; 8.195)	49.084 (1)	<0.001
Without chronic hypertension	7,644	1.000			
**Without gestational hypertension**
Anemia	5,095	0.234	(0.146; 0.374)	36.537 (1)	<0.001
Without anemia	34,217	1.000			
**Gestational hypertension**
Anemia	66	1.879	(0.750; 4.705)	1.813 (1)	0.178
Without anemia	834	1.000			
**Anemia**
Gestational hypertension	66	26.344	(9.775; 70.993)	41.827 (1)	<0.001
Without gestational hypertension	5,095	1.000			
**Without anemia**
Gestational hypertension	834	3.084	(2.240; 4.245)	47.728 (1)	<0.001
Without gestational hypertension	34,217	1.000			

Bonferroni-adjusted significance level (α/4): 0.0125.

Pregnant women without gestational hypertension and with anemia have less chance (odds 0.234; 95% CI: 0.146–0.374) of developing preeclampsia compared to non-anemic gestational hypertensive pregnant women. However, among pregnant women with gestational hypertension, anemia did not increase their likelihood (not significant) of developing preeclampsia.

The frequency of cases that were initially admitted as gestational hypertension and progressed to preeclampsia among pregnant women with anemia, showed an increase odd of developing preeclampsia by 26-fold (odds 26.344; 95% CI: 9.775–70.993, *p* < 0.001) compared to those not as an underlying condition. While among pregnant women without anemia, gestational hypertension led to only three times (odds 3.084; 95% CI: 2.240–4.245, *p* < 0.001) of developing preeclampsia compared to those without gestational hypertension.

### Preeclampsia birth outcomes

Table 4 demonstrates variables on birth outcomes that were significantly associated to preeclampsia. There was a higher prevalence of premature delivery (67.7%), cesarean delivery (70.7%), low birth weight (48.5%), 5-min Apgar score <7 (18.1%), and NICU admission (19.8%) among pregnant women who developed preeclampsia compared to those who did not. Preeclampsia complicated pregnancies had an ~6 times higher likelihood of preterm delivery (adjOR 6.21; 95% CI: 5.24, 7.36), seven times higher likelihood of low-birth weight babies (adjOR 7.87; 95% CI: 6.69, 9.27), eight times greater risk of NICU admission (adjOR 8.78; 95% CI: 7.12, 10.83) and three times greater odds of low 5-min Apgar scores (adjOR 3.16; 95% CI: 2.13, 4.68). Preeclamptic pregnant women were also four times more likely to undergo cesarean delivery (adjOR 3.99; 95% CI: 3.35, 4.75).

**Table 4:** Association of preeclampsia and adverse birth outcomes.

**Variable**	**Preeclampsia *n* (%)**	**No *n* (%)**	**Adjusted OR**	**(95% CI)**	***X*^2^ STAT (*df*)^a^**	***P-*value ^a^**
**Premature delivery**
Yes	434 (67.7)	207 (32.3)	6.214	(5.244; 7.364)	477.430 (1)	<0.001
No	9,474 (23.9)	30,097 (76.1)	1.000			
**Route of delivery**
LSCS	453 (3.43)	12,761 (96.57)	3.987	(3.348; 4.748)	276.634 (1)	<0.001
Vaginal (including instrumental)	188 (0.70)	26,810 (99.30)	1.000			
**Low birth weight**
Yes	311 (48.5)	330 (51.5)	7.873	(6.687; 9.271)	527.289 (1)	<0.001
No	4,117 (10.4)	35,454 (89.6)	1.000			
Mean (Kg) (Standard deviation)	2.465 (SD 0.470)	3.017 (SD 0.755)	0.363	(0.319; 0.312)		<0.001
**Apgar score at 5 min** ** <7**
Yes	116 (18.1)	207 (32.3)	3.158	(2.130; 4.683)	28.247 (1)	<0.001
No	5,565 (14.1)	30,097 (76.1)	1.000			
**NICU admission**
Yes	127 (19.8%)	514 (80.2)	8.778	(7.115; 10.830)	283.577 (1)	<0.001
No	1,066 (2.7)	38,505 (97.3)	1.000			

LSCS, Lower segment Cesarean section.

## Discussion

We found that the prevalence of preeclampsia (1.6%) was consistent with that of a previously reported study at a tertiary academic center in the East Coast of Peninsular Malaysia (1.4%) (4). The prevalence in our center was slightly lower than the global incidence of preeclampsia (2.16%) (24). These differences might be due to variation in maternal age of our cases, socioeconomic background (living in urban setting area), ethnicity (majority were Malay), and enhancement of the Malaysian health care delivery system (25). Preeclampsia prevalence is diverse due to differences in sociodemographic profiles, genetic composition, and geographical areas, as evidenced by previous studies (24, 26, 27).

Malaysia has implemented several maternal health programs in promoting and preventing risk of maternal morbidity and mortality. The Ministry of Health Malaysia introduced an excellent maternal and child health service in the 1950 s and a safe motherhood approach in the 1980 s (28–30). The continuum of care includes pre-pregnancy care, which provides early screening and interventions to minimize preeclampsia risk, such as provision of family planning services for high-risk groups, and preventive public health screening programs in primary health centers for early identification and management of preeclampsia (29). Early diagnosis of hypertension among women followed by pre-conceptional counseling helps mitigate risk of development of complications. Preeclampsia screening and prophylaxis through essential obstetric care are clearly described in the guidelines for healthcare providers (10, 11). Most women in Malaysia depend on free, publicly funded maternal health care (29). Furthermore, improved provision of quality services and access with removal of barriers to health care usage have resulted in better maternal and child health surveillance (25, 29, 31, 32). The surveillance information is beneficially used for formulating intervention strategies to reduce maternal and child deaths, address critical gaps in the health system, and eliminate disparities between different groups through providing special attention to poor and disadvantaged populations, such as people living in rural areas who face logistic barriers (29–31, 33). The maternal mortality ratio in the last 10 years (2010–2020) shows a plateau with ranges from 21 to 24 maternal deaths per one hundred thousand live births (34). Postpartum hemorrhage (17.5%) was recorded as the principal causes of maternal death in 2019 in Malaysia followed by obstetric embolism (12.6%), eclampsia (9.7%), ectopic pregnancy (5.8%) and puerperal sepsis (5.8%). While in 2020, the main cause of maternal death was obstetric embolism (18.8%) followed by postpartum hemorrhage (16.2%), gestational hypertension with significant proteinuria (6.8%), ectopic pregnancy (6.0%) and eclampsia (6.0%). Our center recorded similar pattern which mainly due to direct cause of maternal death (35).

In this study, nulliparity, previous history of preeclampsia, multiple pregnancy, advanced maternal age–chronic hypertension interaction, and anemia–gestational hypertension interaction, were significant factors associated with increased risks of preeclampsia, which are consistent with previous findings (16, 36, 37). Preeclampsia is a common syndrome with uncertain roots that are linked to multiple factors, making public health prevention strategies thereof an endless challenge.

Advanced maternal age, i.e., age ≥35 years at the time of delivery, is a risk factor for preeclampsia and other severe maternal and perinatal adverse pregnancy outcomes resulting in higher morbidity and healthcare costs (38, 39). In our study, 19.5% of the patients were of advanced maternal age, higher than the prevalence estimated by the World Health Organization Multi-Country Survey on Maternal and Newborn Health (12.3%) (40) but lower than that of Norway (33.4%) and Japan (31.1%) (37, 38), and comparable with that of Taiwan (19.1%) (41) and the United Kingdom (18.2%) (42).

In many countries, including Malaysia, maternal age at first delivery has been steadily increasing (39, 43–45). Pregnant women with advanced maternal age were more likely to have a higher incidence of pre-existing diabetes and chronic hypertension, higher BMI, and more assisted conception or fertility treatment (39). Most patients in the sample were Malay (68.6%), who typically start families after marriage as influenced by the Malay culture and Islamic beliefs (46). A recent phenomenon is that an increasing number of Malaysian women delay marriage and motherhood into their late 30 s as they invest time pursuing education and career goals before starting a family (46, 47). This social trend is a result of better gender equality and expanded opportunities for women, increased women's education and labor market participation, changes in life values, partnership, behavior, culture and beliefs, economic insecurity, the availability of effective contraception and assisted reproductive technologies, and the absence of supportive family units (46, 47).

Evidence indicates that some social policies can be effective for counter balancing delayed parenthood (47). Massive postponement is attributable to the conflict between optimal biological period for motherhood and obtaining additional education and building a career. A growing amount of literature demonstrates that female employment and parenting can be combined when policy intervention facilitates the reduction of work–family conflict (47). Women should be well-informed of the risks of advanced maternal age and individual solutions should be complemented by a public health approach, including promoting women to start family when reaching the middle reproductive age (25–29 years) (47). However, our study has limited information on the proportion of ethnicity, educational level and employment status for us to assess the relationship between these factors and preeclampsia. A multi-site study may help in determining the socio demographic association better and be better generalizable to the population of interest.

Multiple pregnancy increases the likelihood of preeclampsia. Our findings support the different pathophysiological pathways of preeclampsia among women with multiple pregnancies as compared to singleton pregnancies (48). The pathway observed in multiple pregnancies is secondary to intraplacental malperfusion and mechanical restrictions. Malperfusion and hypoxia occur when the growing placenta reaches its functional limit (48). Instead of the woman's underlying cardiovascular phenotype, preeclampsia in multiple pregnancy may occur due to the higher demands of a larger placenta on the maternal cardiovascular system, higher levels of circulating placental markers, and excessive placenta-shed inflammatory factors (49, 50). A large cohort study in a Swedish population observed a significantly higher risk of future cardiovascular diseases among preeclampsia-complicated singleton pregnancies compared with singleton pregnancies without preeclampsia and multiple pregnancy with or without preeclampsia (49).

The present study revealed that early-onset chronic hypertension (age <35 years) amplified the odds of developing preeclampsia 15-fold in contrast to that among advanced-maternal age women (age ≥35 years), where chronic hypertension increased the odds of developing preeclampsia by only 5-fold as compared to advanced-maternal age women without chronic hypertension. In the present study, 1.1% of patients had chronic hypertension. The National Health and Morbidity Survey (NHMS) 2019 reported a high prevalence of hypertension (30%) in the Malaysian population that increased with age: 0.4% among women aged 20–24 years and 19.9% among women aged 40–44 years (51). The NHMS 2019 reported that nearly half of the patients were unaware that they had hypertension (51). Therefore, it is expected that many Malaysian women will become pregnant without knowing that they have hypertension, which would hinder pre-pregnancy care intervention for controlling high blood pressure, as well as prophylactic mitigation of risk of development of preeclampsia. Therefore, we suggest that pre-pregnancy care screening for hypertension should be strengthened among women planning to conceive. The major risk factors for hypertension and obesity, for which prevalence is also increasing, complicate preeclampsia intervention in Malaysia (51). Non-Communicable Diseases (NCD) control requires integrated action through all major parts of society that impact health. Nonetheless, many countries have not established system-wide efforts to improve the social determinants of health, e.g., early childhood education and parenting skills, education and lifelong learning, working and employment conditions, poverty reduction and ensuring a healthy standard of living, housing and the environment, and prevention of ill health (52).

In 2016, the Ministry of Health Malaysia developed the National Strategic Plan on NCD to tackle the growing burden of NCDs in Malaysia. The key focus of the Strategic Plan included cardiovascular disease, diabetes, and NCD risk factors, i.e., tobacco use, unhealthy diet, physical inactivity, and harmful use of alcohol. One of the main objectives of this strategy was to strengthen national capacity, leadership, governance, multisectoral action, and partnerships to hasten the Malaysian response for preventing and controlling NCDs (53). There is a crucial need to involve the whole-of-government approach. Nevertheless, the persisting challenge is that agencies outside of healthcare view NCDs as a “health issue.” As such, efficient handover of obligations and accountability for policy movements in addressing the wider elements of NCDs in Malaysia is poor (51).

In the present study, anemia was detected in 12.8% of patients, which is lower than that reported by national study NHMS 2019 (29.9%) (51). A recent systematic review based on published research in Malaysia found that the overall prevalence of anemia in pregnancy ranged from 19.3 to 57.4%, while the prevalence of iron deficiency in pregnancy ranged from 31.6 to 34.6% (54). Lower prevalence of anemia in our center may be due to selecting only single tertiary center where most patients were Malay and of high socioeconomic status living in urban setting of greater Kuala Lumpur. The NHMS 2019 detected higher prevalence of anemia especially among Indians and people of low socioeconomic status (51). Our study found low prevalence of anemia present in preeclampsia cases (3.7%). Many studies have reported that woman with anemia is less likely to develop preeclampsia (55–57).

In the absence of gestational hypertension, anemia reduced the probability of developing preeclampsia, which is in accordance with previous findings (22, 56–59). However, another study reported the significant finding of severe anemia, but not mild/moderate anemia, being a preeclampsia risk factor (60). The increased occurrence of preeclampsia in pregnant women with high hemoglobin levels are the toxic effects of heme sedimentation on the vascular endothelium (61). Iron plays a catalyzing function in the production of reactive oxygen species that leads to oxidative stress, a recognized component of the preeclampsia pathogenesis (62, 63). This supports the premise that raised iron and ferritin levels are associated with greater preeclampsia risk (64, 65). We suspect the possibility of excess iron supplementation among anemic pregnant women in the present study (64, 66). However, we did not examine the iron and ferritin levels to support this statement; we merely discovered an association between anemia and preeclampsia. A cross-sectional design using retrospective data in the study led to the lack of a cause–effect relationship.

We did not show a predicted model for preeclampsia as we identified the interaction between the independent variables (gestational age^*^chronic hypertension and anemia^*^gestational hypertension) on preeclampsia. As we are aware, both conditions produced the same effect with different intensities between subgroups of the variables that has influence on preeclampsia.

Anemic pregnant women initially diagnosed with gestational hypertension showed a 25 times risk for preeclampsia than non-anemic pregnant women. A younger pregnant woman (<35 years) with a history of chronic hypertension has a 14 times higher risk for preeclampsia compared to an older pregnant woman (≥35 years). Hypertension in pregnancy is thought to be composed of various diseases that occur through the confluence of both genetic and acquired factors. The prevalence of hypertension is increasing among the younger age group, possibly linked to genetic factors and lifestyle changes (14, 15, 22). Previous studies highlighted that chronic hypertension and high maternal age increased the risk of preeclampsia (48–51). A higher risk for preeclampsia in a young adult borne by a mother with hypertension in pregnancy may be attributed to a shared environment or to shared genetic factors with the mother (67). A bigger sample size assessing the presence of multiple co-morbidities using multi-study sites may help to determine the association better and generalization to the population of interest. However, in a preventive care plan, it is necessary to ensure mothers with pre-existing chronic hypertension and anemia to come to the pre-pregnancy clinic for appropriate management of their hypertension and to educate them on the possibility of preeclampsia. Early detection and early management of anemia, especially when co-existing with hypertension, will help prevent the development of preeclampsia (60).

Our study found that the preeclampsia was significantly associated with a higher prevalence of poor pregnancy outcomes, including preterm delivery [adjusted OR (adjOR) 6.214], low birth weight (adjOR 6.687), NICU admission (adjOR 8.778), cesarean delivery (adjOR 3.987), and 5-min Apgar score <7 (adjOR 3.158). Our findings similar with previous studies, where a higher incidence of adverse pregnancy outcomes was reported among women with preeclampsia (2, 68, 69). Therefore, preeclamptic women occupy greater levels of healthcare services and hence consume significantly higher healthcare costs (68, 69). In the USA, the 2012 preeclampsia healthcare cost estimates within the first 12 months of delivery for mothers and infants was US$2.18 billion (70) and the mean maternal and infant medical care costs of preeclampsia (US$41,790) were significantly higher compared to that of non-preeclampsia (US$13,187) (71). The economic burden of preeclampsia healthcare is high, the leading cost drivers being infant healthcare costs associated with preterm delivery and greater adverse outcomes (69–72). A recent meta-analysis suggested that an effective intervention for preventing preeclampsia and preterm delivery is early (<16 weeks of gestation) introduction of prophylactic low-dose aspirin (≥100 mg) for high-risk groups (73). This strategy is well-practiced in our Malaysian antenatal care using hypertension management guideline (10).

The retrospective nature of our study led to some limitations. We could not investigate several important risk factors for preeclampsia, including infertility treatment, pregnancy interval, change of sexual partner, alcohol intake, and smoking behavior (74). We used secondary administrative data that were not originally collected for research purposes; thus, the data may have been exposed to bias such as data inaccuracy, missing and incomplete data per variable, and inadequate details in the electronic database. There is a possibility that cases were not always reported adequately. This is consistent with other studies that used electronic database information (75, 76). Our study was conducted at a single tertiary healthcare center; hence the data may not be representative of outcomes in smaller facilities or in other communities outside the research setting, i.e., there is a lack of external validity. Therefore, future study may need to consider having a bigger sample size by using multi centers study to validate and predict better model that able to generalized to the national population with more information added such as attended pre-pregnancy care, socio-economic status, lifestyles and further validate clinical risk factors in the considered population.

## Conclusions

Preeclampsia was significantly associated with the maternal characteristics of nulliparity, history of preeclampsia, multiple pregnancy, and advanced maternal age. A significant interaction was found between chronic hypertension and anemia, as well as between gestational hypertension and maternal age in predicting the incidence of preeclampsia. Although the prevalence of preeclampsia is relatively low, it imposes a higher burden on the healthcare system in managing preeclampsia-related adverse birth outcomes, such as preterm delivery, cesarean delivery, low birth weight, and the increased need for NICU admission. Therefore, it is important to identify women at risk of developing preeclampsia in the effort to prevent adverse birth outcomes through pre-pregnancy counseling for those with chronic hypertension, and anemia, and to promote logical family planning for women at advanced maternal age. Enhancing awareness and healthcare education on safe motherhood should be an ongoing agenda targeting both mothers and their community. Future studies should utilize multi-center data bases for generalization to a diverse population with inclusion of several important social behavioral factors.

## Data availability statement

The data analyzed in this study is subject to the following licenses/restrictions: Permission to use the dataset was obtained from Department of Obstetrics HCTM through the Ethical Committee approval [UKM Ethics Committee (FF-2019-371)]. Requests to access these datasets should be directed to sepukm@ukm.edu.my (UKM Ethics Committee).

## Author contributions

NA, RS, and ZM designed the research methodology, wrote the manuscript, and interpret and validated the results. NA run the analyzed. All authors contributed to the article and approved the submitted version.

## Funding

This research was funded by a UKM Fundamental Grant (FF-2019-371) and Matching Grant (FF-2019-371/1).

## Conflict of interest

The authors declare that the research was conducted in the absence of any commercial or financial relationships that could be construed as a potential conflict of interest.

## Publisher's note

All claims expressed in this article are solely those of the authors and do not necessarily represent those of their affiliated organizations, or those of the publisher, the editors and the reviewers. Any product that may be evaluated in this article, or claim that may be made by its manufacturer, is not guaranteed or endorsed by the publisher.

## References

[1] Say L Chou D Gemmill A Tunçalp Ö Moller A-B Daniels J. Global causes of maternal death: a WHO systematic analysis. Lancet Global Health. (2014) 2:e323–e33. 10.1016/S2214-109X(14)70227-X 25103301

[2] Kongwattanakul K Saksiriwuttho P Chaiyarach S Thepsuthammarat K. Incidence, characteristics, maternal complications, and perinatal outcomes associated with preeclampsia with severe features and HELLP syndrome. Int J Womens Health. (2018) 10:371–7. 10.2147/IJWH.S168569 30046254 PMC6054275

[3] Abalos E Cuesta C Grosso AL Chou D Say L. Global and regional estimates of preeclampsia and eclampsia: a systematic review. Eur J Obstet Gynecol Reprod Biol. (2013) 170:1–7. 10.1016/j.ejogrb.2013.05.005 23746796

[4] Mahamooth DMIJ. A retrospective Study on Pre-Eclampsia in Hospital Universiti Sains Malaysia, Kelantan: Pusat Pengajian Sains Perubatan, Universiti Sains Malaysia. (2018). Available online at: http://eprints.usm.my/46172/2/Dr.%20Mas%20Irfan%20Jaya%20Mahamooth-24%20pages.pdf (accessed September 30, 2022).

[5] Mol BWJ Roberts CT Thangaratinam S Magee LA de Groot CJM Hofmeyr GJ. Pre-eclampsia. Lancet. (2016) 387:999–1011. 10.1016/S0140-6736(15)00070-7 26342729

[6] Anderson U Olsson M Kristensen K Åkerström B Hansson S. Biochemical markers to predict preeclampsia. Placenta. (2012) 33:S42–S7. 10.1016/j.placenta.2011.11.021 22197626

[7] Brown MA Magee LA Kenny LC Karumanchi SA McCarthy FP Saito S. Hypertensive disorders of pregnancy: ISSHP classification, diagnosis, and management recommendations for international practice. Hypertension. (2018) 72:24–43. 10.1161/HYPERTENSIONAHA.117.10803 29899139

[8] Von Dadelszen P Magee LA. Preventing deaths due to the hypertensive disorders of pregnancy. Best Pract Res Clin Obstet Gynaecol. (2016) 36:83–102. 10.1016/j.bpobgyn.2016.05.005 27531686 PMC5096310

[9] Perez-Cuevas R Fraser W Reyes H Reinharz D Daftari A Heinz CS. Critical pathways for the management of preeclampsia and severe preeclampsia in institutionalised health care settings. BMC Pregnancy Childbirth. (2003) 3:6. 10.1186/1471-2393-3-6 14525621 PMC270024

[10] Ministry of Health Malaysia. 5th Edition Clinical Practice Guideline Management of Hypertension. (2018). Available online at: http://www.acadmed.org.my/index.cfm?menuid=67 (accessed September 30, 2022).

[11] Malaysia NTCCEIMD,. Training Manual Hypertensive Disorders in Pregnancy Revised 2018. Ministry of Health Malaysia: Family Health Development Division Ministry of Health, Malaysia. (2018). Available online at: https://fh.moh.gov.my/v3/index.php/component/jdownloads/send/18-sektor-kesihatan-ibu/235-training-manual-on-hypertensive-disorders-in-pregnancy?Itemid=0 (accessed September 30, 2022).

[12] Gestational Hypertension and Preeclampsia: ACOG Practice Bulletin Number 222. Obstet Gynecol. (2020) 135:e237–60. 10.1097/AOG.0000000000003891 32443079

[13] National Collaborating Centre for Women's and Children's Health (UK). Hypertension in Pregnancy: The Management of Hypertensive Disorders During Pregnancy. London: RCOG Press (2010). 22220321

[14] Quan L-M Xu Q-L Zhang G-Q Wu L-L Xu H. An analysis of the risk factors of preeclampsia and prediction based on combined biochemical indexes. Kaohsiung J Med Sci. (2018) 34:109–12. 10.1016/j.kjms.2017.10.001 29413226 PMC11915607

[15] Sohlberg S Stephansson O Cnattingius S Wikström A-K. Maternal body mass index, height, and risks of preeclampsia. Am J Hypertens. (2012) 25:120–5. 10.1038/ajh.2011.175 21976280

[16] Khader YS Batieha A Al-Njadat RA. Hijazi SaS. Preeclampsia in Jordan: incidence, risk factors, and its associated maternal and neonatal outcomes. J Maternal Fetal Neonatal Med. (2018) 31:770–6. 10.1080/14767058.2017.1297411 28274172

[17] Osungbade KO Ige OK. Public health perspectives of preeclampsia in developing countries: implication for health system strengthening. J Pregnancy. (2011) 2011:481095. 10.1155/2011/481095 21547090 PMC3087154

[18] Xiao J Shen F Xue Q Chen G Zeng K Stone P. Is ethnicity a risk factor for developing preeclampsia? An analysis of the prevalence of preeclampsia in China. J Human Hypertension. (2014) 28:694–8. 10.1038/jhh.2013.148 24430700

[19] López-Jaramillo P Pradilla LP Castillo VR Lahera V. Socioeconomic pathology as a cause of regional differences in the prevalence of metabolic syndrome and pregnancy-induced hypertension. Rev Española Cardiol. (2007) 60:168–78. 10.1016/S1885-5857(07)60129-7 17338881

[20] Universiti Kebangsaan Malaysia,. Laporan Tahunan Pusat Perubatan (UKMMC Annual Report) UKM 2015. (2015). Available online at: https://hctm.ukm.my/wp-content/uploads/2021/04/buku-laporan-2015.pdf (accessed September 30, 2022).

[21] Kementerian Kesihatan Malaysia,. Garis Panduan Senarai Semak Bagi Penjagaan Kesihatan Ibu Mengikut Sistem Kod Warna. (2020). Available online at: https://fh.moh.gov.my/v3/index.php/component/jdownloads/send/18-sektor-kesihatan-ibu/771-garis-panduan-senarai-semak-bagi-penjagaan-kesihatan-ibu-mengikut-sistem-kod-warna-2020-compressed?Itemid=0 (accessed September 30, 2022).

[22] Paré E Parry S McElrath TF Pucci D Newton A Lim KH. Clinical risk factors for preeclampsia in the 21st century. Obstet Gynecol. (2014) 124:763–70. 10.1097/AOG.0000000000000451 25198274

[23] Bland JM Altman DG. Multiple significance tests: The Bonferroni method. BMJ. (1995) 310:170. 10.1136/bmj.310.6973.170 7833759 PMC2548561

[24] Abalos E Cuesta C Carroli G Zahida Q Widmer M Vogel J. Pre-eclampsia, eclampsia and adverse maternal and perinatal outcomes: a secondary analysis of the world health organization multicountry survey on maternal and newborn health. BJOG Int J Obstet Gynaecol. (2014) 121:14–24. 10.1111/1471-0528.12629 24641531

[25] Yeoh PL Hornetz K Dahlui M. Antenatal care utilisation and content between low-risk and high-risk pregnant women. PLoS ONE. (2016) 11:e0152167. 10.1371/journal.pone.0152167 27010482 PMC4807004

[26] Bilano VL Ota E Ganchimeg T Mori R Souza JP. Risk factors of pre-eclampsia/eclampsia and its adverse outcomes in low- and middle-income countries: a WHO secondary analysis. PLoS ONE. (2014) 9:e91198. 10.1371/journal.pone.0091198 24657964 PMC3962376

[27] Moungmaithong S Wang X Tai AST Feng Q Sahota D Leung TY. First trimester screening for preeclampsia: an asian perspective. Maternal Fetal Med. (2021) 3:116–23. 10.1097/FM9.0000000000000101

[28] Malaysian Healthcare Performance Unit Malaysian Health at a Glance: 2018 Ministry Ministry of Health Malaysia: Putrajaya. Available online at: https://www.moh.gov.my/moh/penerbitan/MYHAAG2018.pdf (accessed September 30, 2022).

[29] Yadav H. A review of maternal mortality in Malaysia. IeJSME. (2012) 6:S142–S51. 10.56026/imu.6.Suppl1.S142

[30] Liljestrand J Pathmanathan I. Reducing maternal mortality: can we derive policy guidance from developing country experiences? J Public Health Policy. (2004) 25:299–314. 10.1057/palgrave.jphp.3190030 15683067

[31] World Health Organization. Regional Office for the Western Pacific. Malaysia Health System Review. WHO Regional Office for the Western Pacific. (2012). Avaiable online at: https://apps.who.int/iris/handle/10665/206911 (accessed September 30, 2022).

[32] Loganathan T Rui D Ng C-W Pocock NS. Breaking down the barriers: Understanding migrant workers' access to healthcare in Malaysia. PLoS ONE. (2019) 14:e0218669. 10.1371/journal.pone.0218669 31269052 PMC6608924

[33] Al-Tairi ANQ Isa ZM Ghazi HF. Risk factors of preeclampsia: a case control study among mothers in Sana'a, Yemen. J Public Health. (2017) 25:573–80. 10.1007/s10389-017-0825-0

[34] DOSM. Statistics and Causes of Death Malaysia. Available online at: https://www.dosm.gov.my/v1/index.php?r=column/cthemeByCat&cat=401&bul_id=R3VrRUhwSXZDN2k4SGN6akRhTStwQT09&menu_id=L0pheU43NWJwRWVSZklWdzQ4TlhUUT09 (accessed September 30, 2022).

[35] Abd Rahman R Ismail NM Yassin MA Sulaiman AS. Comparative review of fourteen years maternal mortality in achieving MDG5 in Malaysia and UKMMC. Malaysian J Public Health Med. (2013) 13:59–63. Available online at: https://www.researchgate.net/publication/284371779_SHORT_REVIEW_OF_FOURTEEN_YEARS_MATERNAL_MORTALITY_IN_ACHIEVING_MDG5_IN_UKMMC

[36] Robillard P-Y Dekker G Chaouat G Scioscia M Iacobelli S Hulsey TC. Historical evolution of ideas on eclampsia/preeclampsia: a proposed optimistic view of preeclampsia. J Reprod Immunol. (2017) 123:72–7. 10.1016/j.jri.2017.09.006 28941881 PMC5817979

[37] Laine K Murzakanova G Sole KB Pay AD Heradstveit S Räisänen S. Prevalence and risk of pre-eclampsia and gestational hypertension in twin pregnancies: a population-based register study. BMJ Open. (2019) 9:e029908. 10.1136/bmjopen-2019-029908 31278106 PMC6615795

[38] Tyas BD Lestari P Akbar MIA. Maternal perinatal outcomes related to advanced maternal age in preeclampsia pregnant women. J Family Reprod Health. (2019) 13:191. 10.18502/jfrh.v13i4.2646 32518569 PMC7264866

[39] Lamminpää R Vehviläinen-Julkunen K Gissler M Heinonen S. Preeclampsia complicated by advanced maternal age: a registry-based study on primiparous women in Finland 1997–2008. BMC Pregnancy Childbirth. (2012) 12:1–5. 10.1186/1471-2393-12-47 22687260 PMC3495042

[40] Laopaiboon M Lumbiganon P Intarut N Mori R Ganchimeg T Vogel J. Advanced maternal age and pregnancy outcomes: a multicountry assessment. BJOG Int J Obstet Gynaecol. (2014) 121:49–56. 10.1111/1471-0528.12659 24641535

[41] Liou J-D Hsu J-J Lo L-M Chen S-F Hung T-H. Advanced maternal age and adverse perinatal outcomes in an Asian population. Eur J Obstet Gynecol Reproduct Biol. (2010) 148:21–6. 10.1016/j.ejogrb.2009.08.022 19773110

[42] Kenny LC Lavender T McNamee R O'Neill SM Mills T Khashan AS. Advanced maternal age and adverse pregnancy outcome: evidence from a large contemporary cohort. PLoS ONE. (2013) 8:e56583. 10.1371/journal.pone.0056583 23437176 PMC3577849

[43] Rashed H Awaluddin S Ahmad NA Supar N Lani Z Aziz F. Advanced maternal age and adverse pregnancy outcomes in Muar, Johor, Malaysia. Sains Malays. (2016) 45:1537–42. Available online at: http://journalarticle.ukm.my/10299/1/15%20Herny%20Erdawati.pdf

[44] Kyozuka H Fujimori K Hosoya M Yasumura S Yokoyama T Sato A. The effect of maternal age at the first childbirth on gestational age and birth weight: the Japan environment and children's study (JECS). J Epidemiol. (2018) 2018:JE20170283. 10.2188/jea.JE20170283 30078812 PMC6445800

[45] Beaujouan É Toulemon L. European countries with delayed childbearing are not those with lower fertility. Genus. (2021) 77:1–15. 10.1186/s41118-020-00108-033456069

[46] Rozita I. Hassan Z. Never-married malay muslim women in modernised malaysia: are they rejecting marriage? Global Studies J. (2009) 2:45–56. 10.18848/1835-4432/CGP/v02i02/41004

[47] Mills M Rindfuss RR McDonald P. Te Velde E, Reproduction E, Force ST. Why do people postpone parenthood? Reasons and social policy incentives. Human Reprod Update. (2011) 17:848–60. 10.1093/humupd/dmr026 21652599 PMC3529638

[48] Staff AC. The two-stage placental model of preeclampsia: an update. J Reprod Immunol. (2019) 134:1–10. 10.1016/j.jri.2019.07.004 31301487

[49] Bergman L Nordlöf-Callbo P Wikström AK Snowden JM Hesselman S Bonamy AKE. Multi-fetal pregnancy, preeclampsia, and long-term cardiovascular disease. Hypertension. (2020) 76:167–75. 10.1161/HYPERTENSIONAHA.120.14860 32475315 PMC7289126

[50] Bdolah Y Lam C Rajakumar A Shivalingappa V Mutter W Sachs BP. Twin pregnancy and the risk of preeclampsia: bigger placenta or relative ischemia? Am J Obstet Gynecol. (2008) 198:428. 10.1016/j.ajog.2007.10.783 18191808

[51] Institute for Public Health (IPH) National National Institutes of Health Ministry Ministry of Health Malaysia. 2020. National Health and Morbidity Survey (NHMS) 2019: Vol. I: NCDs – Non-Communicable Diseases: Risk Factors and other Health Problems ISBN: e978-967-18159-2-2. Available online at: https://iku.moh.gov.my/images/IKU/Document/REPORT/NHMS2019/Report_NHMS2019-NCD_v2.pdf (accessed September 30, 2022).

[52] Marmot M Bell R. Social determinants and non-communicable diseases: time for integrated action. BMJ. (2019) 364:l251. 10.1136/bmj.l251 30692093 PMC6348404

[53] Ministry of Health. National Strategic Plan for Non-Communicable Disease (NSPNCD) 2016-2025. (2016). Available online at: https://www.moh.gov.my/moh/resources/Penerbitan/Rujukan/NCD/National%20Strategic%20Plan/FINAL_NSPNCD.pdf (accessed September 30, 2022).

[54] Abd Rahman R Idris IB Isa ZM Rahman RA Mahdy ZA. The prevalence and risk factors of iron deficiency anemia among pregnant women in malaysia: a systematic review. Front Nutr. (2022) 9:847693. 10.3389/fnut.2022.847693 35495961 PMC9051477

[55] Seely EW Ecker J. Chronic hypertension in pregnancy. Circulation. (2014) 129:1254–61. 10.1161/CIRCULATIONAHA.113.003904 24637432

[56] Wang C Lin L Su R Zhu W Wei Y Yan J. Hemoglobin levels during the first trimester of pregnancy are associated with the risk of gestational diabetes mellitus, pre-eclampsia and preterm birth in Chinese women: a retrospective study. BMC Pregnancy Childbirth. (2018) 18:263. 10.1186/s12884-018-1800-7 29940874 PMC6020184

[57] Aghamohammadi A Zafari M Tofighi M. High maternal hemoglobin concentration in first trimester as risk factor for pregnancy induced hypertension. Caspian J Intern Med. (2011) 2:194–7. 24024014 PMC3766933

[58] Burton GJ Redman CW Roberts JM Moffett A. Pre-eclampsia: pathophysiology and clinical implications. BMJ. (2019) 366:l2381. 10.1136/bmj.l2381 31307997

[59] Yemane A Teka H Ahmed S Temesgen H Langen E. Gestational hypertension and progression towards preeclampsia in Northern Ethiopia: prospective cohort study. BMC Pregnancy Childbirth. (2021) 21:261. 10.1186/s12884-021-03712-w 33784971 PMC8008690

[60] Ali AA Rayis DA Abdallah TM Elbashir MI Adam I. Severe anaemia is associated with a higher risk for preeclampsia and poor perinatal outcomes in Kassala hospital, eastern Sudan. BMC Res Notes. (2011) 4:311. 10.1186/1756-0500-4-311 21867566 PMC3224576

[61] Balla G Vercellotti GM Muller-Eberhard U Eaton J Jacob HS. Exposure of endothelial cells to free heme potentiates damage mediated by granulocytes and toxic oxygen species. Lab Invest. (1991) 64:648–55. 2030579

[62] Myatt L Webster RP. Vascular biology of preeclampsia. J Thromb Haemostasis. (2009) 7:375–84. 10.1111/j.1538-7836.2008.03259.x 19087223

[63] Rana S Powe CE Salahuddin S Verlohren S Perschel FH Levine RJ. Angiogenic factors and the risk of adverse outcomes in women with suspected preeclampsia. Circulation. (2012) 125:911–9. 10.1161/CIRCULATIONAHA.111.054361 22261192 PMC3319742

[64] Singh L Thakur A Khan FM Misra M Singh S. Prophylactic iron supplementation in pregnancy and its implications in development of preeclampsia: a case control study. J Clin Diagn Res. (2021) 15:BC22–6. 10.7860/JCDR/2021/50696.15530

[65] Kell DB Pretorius E. Serum ferritin is an important inflammatory disease marker, as it is mainly a leakage product from damaged cells. Metallomics. (2014) 6:748–73. 10.1039/C3MT00347G 24549403

[66] Maitra S Mukthapuram A Huligol G Sreelatha G Vishwanath H. Increased serum ferritin and iron levels in preeclampsia. IOSR. (2019) 5:50–2. Available online at: https://www.iosrjournals.org/iosr-jbb/papers/Volume%205,%20Issue%202/Series-1/G0502015052.pdf

[67] Alsnes IV Vatten LJ Fraser A Bjørngaard JH Rich-Edwards J Romundstad PR. Hypertension in pregnancy and offspring cardiovascular risk in young adulthood: Prospective and sibling studies in the hunt study (nord-trøndelag health study) in Norway. Hypertension. (2017) 69:591–8. 10.1161/HYPERTENSIONAHA.116.08414 28223467

[68] Fox R Kitt J Leeson P Aye CY Lewandowski AJ. Preeclampsia: risk factors, diagnosis, management, and the cardiovascular impact on the offspring. J Clin Med. (2019) 8:1625. 10.3390/jcm8101625 31590294 PMC6832549

[69] Maged AM Elsherief A Hassan H Salaheldin D Omran KA Almohamady M. Maternal, fetal, and neonatal outcomes among different types of hypertensive disorders associating pregnancy needing intensive care management. J Maternal Fetal Neonatal Med. (2020) 33:314–21. 10.1080/14767058.2018.1491030 29914278

[70] Fox A McHugh S Browne J Kenny LC Fitzgerald A Khashan AS. Estimating the cost of preeclampsia in the healthcare system: cross-sectional study using data from SCOPE study (Screening for Pregnancy End Points). Hypertension. (2017) 70:1243–9. 10.1161/HYPERTENSIONAHA.117.09499 29084880

[71] Hao J Hassen D Hao Q Graham J Paglia MJ Brown J. Maternal and infant health care costs related to preeclampsia. Obstet Gynecol. (2019) 134:1227–33. 10.1097/AOG.0000000000003581 31764733 PMC6882523

[72] Stevens W Shih T Incerti D Ton TG Lee HC Peneva D. Short-term costs of preeclampsia to the United States health care system. Am J Obstet Gynecol. (2017) 217:237–48. 10.1016/j.ajog.2017.04.032 28708975

[73] Yip KC Luo Z Huang X Lee W Li L Dai C. The role of aspirin dose and initiation time in the prevention of preeclampsia and corresponding complications: a meta-analysis of RCTs. Arch Gynecol Obstet. (2022) 305:1465–79. 10.1007/s00404-021-06349-4 34999942

[74] Shen M Smith GN Rodger M White RR Walker MC Wen SW. Comparison of risk factors and outcomes of gestational hypertension and pre-eclampsia. PLoS ONE. (2017) 12:e0175914. 10.1371/journal.pone.0175914 28437461 PMC5402970

[75] Sutan R Mohamed NE Mahdy ZA Ishak S Shamsuddin K Idris IB. A 5 year trend and predictors of preterm births in single referral centre of the Greater Kuala Lumpur, Malaysia. Int J Pregn Chi Birth. (2018) 4:196–201. 10.15406/ipcb.2018.04.00126

[76] Wang Y Tanbo T Åbyholm T Henriksen T. The impact of advanced maternal age and parity on obstetric and perinatal outcomes in singleton gestations. Arch Gynecol Obstet. (2011) 284:31–7. 10.1007/s00404-010-1587-x 20632182 PMC3112324

